# Analysis of gene expression to predict dynamics of future hypertension incidence in type 2 diabetic patients

**DOI:** 10.1186/s12919-016-0015-z

**Published:** 2016-10-18

**Authors:** Piotr Radkowski, Gracjan Wątor, Jan Skupien, Anna Bogdali, Paweł Wołkow

**Affiliations:** 1 Center for Medical Genomics–OMICRON, Jagiellonian University Medical College, Kraków, Poland; 2 III Chair of Surgery, Jagiellonian University Medical College, Kraków, Poland; 3 Department of Metabolic Diseases, Jagiellonian University Medical College, Kraków, Poland; 4 Department of Internal and Agricultural Medicine, Jagiellonian University Medical College, Kraków, Poland; 5 Department of Pharmacology, Jagiellonian University Medical College, Kraków, Poland

## Abstract

**Background:**

The main focus of the Genetic Analysis Workshop 19 (GAW19) is identification of genes related to the occurrence of hypertension in the cohort of patients with type 2 diabetes mellitus (T2DM). The aim of our study was to predict dynamics of the future hypertension incidence, based on gene expression profiles, systolic and diastolic blood pressure changes in time, sex, baseline age, and cigarette smoking status.

We analyzed data made available to GAW19 participants, which included gene expression profiles of peripheral blood mononuclear cells (PBMCs) from the diabetic members of 20 Mexican American families.

**Methods:**

On the basis of mid blood pressure measurements at several time points, the coefficient of regression (slope) was calculated for each individual. We corrected the slope value in patients treated with antihypertensive medications. Feature preprocessing methods were used to remove highly correlated probes and linear dependencies between them. Subsequently, multiple linear regression model was used to associate gene expression with the regression coefficient calculated for each T2DM patient. Tenfold cross-validation was used to validate the model. We used linear mixed effects model and kinship coefficients to account for the family structure. All calculations were performed in R.

**Results:**

This analysis allowed us to identify 6 well-annotated genes: *RTP4, FXYD6, GDF11, IFNAR1, NOX3,* and *HLA-DQ2,* associated with dynamics of future hypertension incidence. Two of them, *IFNAR1* and *NOX3* were previously implicated in pathogenesis of hypertension.

**Conclusions:**

There is no obvious mechanism that links all detected genes with dynamics of hypertension incidence. Identification of possible connection with hypertension needs further investigation.

## Background

As a result of civilization factors, the incidence of hypertension is increased [1]. Hypertension increases the risk of many life-threatening medical conditions, including stroke and myocardial infarction. This is especially important for patients with various comorbidities which lead to similar medical complications, which is the case in patients with type 2 diabetes mellitus (T2DM) and hypertension. Therefore, it should be useful to identify functional genes responsible for the development of civilization diseases to predict also their progression in time in human population studies [2–4]. Analysis of gene expression may be helpful in this task [3, 5]. Since 1999, microarray-based gene expression analyses have become one of the most popular technologies in transcriptomics studies. Over the last 15 years, numerous studies addressed the use of array expression profiling of mononuclear cells from peripheral blood of patients with various diseases. This great effort was conducted to elucidate molecular background of diseases and simultaneously serve primary diagnosis, differential diagnosis, subclassification, study therapy outcomes, as well as prognostic and predictive biomarkers [6]. One of the most challenging issues in transcriptomics is to understand complex diseases, such as hypertension, especially in combination with T2DM. To understand the occurrence and progression of hypertension it is important to understand gene expression and their correlation with the disease [6]. In future, methods that simplify the evaluation of this molecular signature will be required for routine use in clinical decision making. The main assumption to conduct this type of research is that a set of messenger RNA molecules could indicate future health and/or a pathological condition. In our study, we want to identify predictors of dynamics of future hypertension incidence in patients who already developed T2DM and are at increased risk of cardiovascular complications.

## Methods

The San Antonio Family Heart Study collected phenotype data for 1371 patients and expression data were made available to Genetic Analysis Workshop 19 (GAW19) participants for 647 patients with T2DM [7]. Only 502 patients had both data sets available and were neither hypertensive (systolic blood pressure <140 mmHg, diastolic blood pressure <90 mmHg) nor taking antihypertensive drugs at first visit. Blood was collected for gene expression analysis at the time of first blood pressure measurement. However, only 340 patients had also between 1 and 4 additional blood pressure measurements at various time points (1 to 19 years) after the first visit. Among 340 T2DM patients included in our analysis, 220 remained normotensive throughout the duration of the project, 38 were declared hypertensive based on blood pressure measurements, and 82 were declared hypertensive because they started to use antihypertensive drugs (Table 1).

**Table 1:** Characteristics of the study population

Subgroup	1st	2nd	3rd
Developed hypertension	NoHTN	HTN	HTN
Medications	NoBPM	NoBPM	BPM
Slope (median)	0.4138	1.7714	0.6546
Number (340)	220	38	82
Age (median & range)	27.16 (15.96–78.59)	36.34 (16.04–69.10)	42.97 (19.32–71.29)
MBP (median & range)	89.5 (66–110.5)	96.25 (80–107.5)	97.00 (71–111)

*BPM* blood pressure medication, *HTN* developed hypertension, *MBP* mid blood pressure, *NoBPM* no blood pressure medications, *NoHTN* no developed hypertension

To model future hypertension using gene expression data we applied linear model regression, multiple linear model regression, linear mixed models using kinship matrix, and K-fold cross-validation. All calculations were performed using Rstudio with additional packages like *foreach, doParallel, plyr, reshape2, ggplot2, illuminaHumanv1.db, kinship2, Matrix, coxme, caret, FSelector.* KING software was used to determine kinship coefficients from available genotype data (variant call format [VCF] files).

### Calculation strategy

First we performed data filtering to obtain 340 patients.

Then we calculated the regression coefficient (slope) for each individual using linear regression with MBP (mid blood pressure defined as [systolic blood pressure (SBP) + diastolic blood pressure (DBP)]/2) as a dependent variable and time of subsequent measurements as an independent variable.

The group was divided as described above and we determined the median MBP value for each group.

We adjusted slope of every patient from group 3 (see Table 1) by adding a factor, which is the difference between the medians of the second and third groups.

### Features preprocessing

We removed highly correlated probes and linear dependencies between them to reduce the number of probes to 340. Then we used univariate screen of these probes, to select probes which are associated with a slope at *p* <0.1, using the caret package.

This procedure selected 20 probes with association of expression at the level of *p* <0.1, which were used as predictors, together with age, cigarette smoking, and sex, in a multiple linear regression model.

For the last step of calculations we used K cross-validation for resampling of our model.

For the following step of calculations we used K cross-validation for resampling of our model.

Finally, we calculated kinship coefficient for each pair of individuals within families, using pedigree structure (kinship2 package) or using available genotype data (King software) and used kinship matrix in a linear mixed effects model.

From the initial study group we selected 340 patients without hypertension phenotype. Median values of the MBP slopes were 0.41; 1.77; 0.65 for first, second, and third group, respectively. Size of the consecutive groups was 220, 38, and 82 cases for the first, second, and third group, respectively.

## Results

### Calculation of outcome variable

The first step was to calculate regression coefficient (slope), which represents dynamics of progression to hypertension. We used a linear regression model with mid baseline blood pressure data (MBP defined as [SBP + DBP]/2) as a dependent variable and measurement time data as an independent variable. Then we divided study group into 3 different phenotypes. First group consisted of patients who did not develop hypertension (NoHTN) and who do not take any blood pressure medications (NoBPM); the second group consisted of patients who developed hypertension (HTN) but are not treated for HTN (NoBPM); and the third group consisted of patients with developed HTN who take blood pressure medication (BPM). We determined median for each group (see Table 1).

### Discussion on the distribution mid blood pressure in each group

We checked distribution of MBP within groups. Surprisingly, MBP median value of the second and third groups was significantly higher than that observed in the first group (89.5; 96.25; and 97 mmHg for first, second, and third groups, respectively). This means that T2DM patients who later developed HTN, already had a tendency to have higher blood pressure at baseline, although still within the normal limits. However, based on these differences we could not provide a diagnostic test that supports clinical decision making. We can see the trend, but to create a clinically useful predictor we need a stronger discriminator, such as a set of genes.

We assume that patients from a third group would have a higher blood pressure at later measurements and, in consequence, a higher regression coefficient (slope), provided they do not take any medication. So, we adjusted slope of every patient from group 3 by adding the difference between median of the second and third groups’ slopes.

### Strategy to select gene expression probes to model dynamics of hypertension

Use of more than 20,000 gene expression probes for modeling the response in 340 subjects in the form of a full model or univariate screen of probes does not provide a stable model. Therefore, we used feature preprocessing capabilities implemented in caret package of R to decrease the dimensionality of the data. First, we removed probes with highly correlated expressions, using as the cutoff point for probe removal correlations above 0.75. Subsequently, we removed linear dependencies between the probes. This resulted in reduction of the number of the probes used for further modeling from 20,634 – 340.

To construct the final model of dynamic progression to HTN, starting with preprocessed 340 probes, we performed a univariate screen of all probes with a linear regression model (lm function of R) against the slope. We included in the final model all probes with a *p* value of association of less than 0.1. We also forced into the model environmental covariates: age at the time of first examination, sex, and smoking status. The first predictor variable is age at the time of the first blood pressure measurements, which is identical to the time of blood drawing for gene expression analysis. As expected, the age was substantially higher in the second and third groups, in agreement with the tendency of pressure increase with age (median value 27.16; 36.34; 42.97 for first, second, and third groups, respectively). The next predictors are cigarette smoking and sex, included in the model as recorded during the first visit.

Fitting of these models to perform a univariate screen of all probes, results in selection of 20 probes associated with dynamics of HTN development at *p* value of less than 0.1. Together with 3 environmental variables, the final model includes 23 variables. The multiple R2 for this model is 0.2106, adjusted R2 is 0.1532 with a *p* value of 9.11 × 10^−8^. Seven probes were significantly associated with the slope, these include probes for the following genes: *RTP4, FXYD6, GDF11, IFNAR1, NOX3,* and *HLA-DQA2,* as well as 1 probe with lack of annotation (Table 2).

**Table 2:** List of significantly associated genes after fitting linear model

Number^a^	Probe number	*p* value	Gene name
1	GI_11545889.S	0.01759156	*RTP4*
2	GI_11612654.S	0.02029625	*FXYD6*
3	GI_10863872.S	0.02288918	*NA* ^b^
4	GI_11641418.S	0.02806682	*GDF11*
5	GI_10835182.S	0.03843530	*IFNAR1*
6	GI_11136625.S	0.03981958	*NOX3*
7	GI_11095446.S	0.04552203	*HLA-DQA2*

^a^Number of probes with a *p* value of less than 0.05

^b^Not applicable (NA)

### Validation of the model

We validated this model using 10-fold cross-validation. R2 of the cross-validated full model was 0.1459.

### Testing for the possible family effects

We calculated kinship coefficients for each pair of individuals within the families. Kinship coefficients were calculated in 1 of 2 ways: either using provided pedigree structure (kinship2 package of R) or using genotype data (KING: kinship based inference for GWAS [genome-wide association studies]). Genotype data were available for 200 individuals from our data set. Kinship coefficients calculated with these 2 approaches were nearly identical. No misparenting events, sample mixup, or hidden family structure were detected (data not shown). To avoid decrease in our sample size, we used kinship coefficients calculated from pedigree structure in all further analyses.

To incorporate kinship coefficients in the linear mixed effects model we used the *coxme* package of R with a function *lmekin*. No random effects of family structure were associated with the slope in this model (Table 3).

**Table 3:** List of significantly associated genes after fitting linear mixed effects model using a kinship matrix

Number^a^	Probe number	*p* value	Gene name
1	GI_11545889.S	0.01788168	*RTP4*
2	GI_11612654.S	0.01858857	*FXYD6*
3	GI_10863872.S	0.02286732	*NA* ^b^
4	GI_11641418.S	0.02691985	*GDF11*
5	GI_11136625.S	0.03940994	*NOX3*
6	GI_10835182.S	0.04374117	*IFNAR1*
7	GI_11095446.S	0.04893729	*HLA-DQA2*

^a^Number of probes with a *p* value of less than 0.05

^b^Not applicable (NA)

## Discussion

We performed our analysis so as to identify genes whose expression can predict the dynamics of future HTN incidence in T2DM patients. We identified 6 well-annotated genes (*RTP4, FXYD6, GDF11, IFNAR1, NOX3,* and *HLA-DQ2*) and 1 probe that does not correspond to any gene, which fulfill that criterion and remain significantly associated with our outcome after cross-validation.

However, we have to stress that the data set provided for analysis has several limitations. Analysis of gene expression has shifted in the past few years from microarrays to next-generation sequencing of RNA. New methods provide better dynamic range and allow the finding of unexpected transcripts. Even the type of microarrays used in this study is suboptimal; exon microarrays could allow us to discriminate between alternative transcripts, which may play an important role in development of HTN.

The study group is not very big by current standards. Nowadays, microarray experiments involve often tens of thousands of subjects. Taken together, the power to identify true associations of gene expression with HTN, if they exist at all, is diminished. The environmental data are quite scarce, too - only age at baseline, smoking status, and sex are available. This group of patients is probably quite heterogenous. We have no information about age of onset of diabetes, duration of diabetes, current body mass index, glycemic control, or pharmacotherapy at baseline and its changes during the study. We can, for example, imagine how profound an impact on gene expression insulin therapy and dose used may have.

Another open question is whether peripheral blood mononuclear cells (PBMCs) are an adequate source of information in this context. Maybe a liver, adipose tissue, muscle or pancreatic biopsy, being closer to the pathogenesis of diabetes, could be a better source of information than PBMCs? Possibly a renal tissue would be more informative for HTN? Unfortunately, these tissue fragments are very hard to obtain. On the other hand, mononuclear cells are involved in the development of inflammation, which is one of the causal pathways of HTN. Nevertheless, this data set provides an interesting opportunity to check the modeling strategy in prediction of the events distant in time.

We decided to construct the outcome variable that captured scattered-in-time information about blood pressure measurements. The slope of blood pressure changes allows us to deal efficiently with the situation in which time of consecutive patient visits and blood pressure changes was quite irregular. Our concern was that the slope of blood pressure changes was rather flat among patients identified as hypertensive based on antihypertensive treatment they used - quite efficiently for the majority of them, as the data show. We decided that the best available correction would be to adjust their slope by a factor seen in patients identified as hypertensive based on their blood pressure.

Another difficulty we encountered was a large number of features (more than 20,000) to be analyzed with a rather modest number of subjects (only 340). To avoid instability of the model created in such a situation, we implemented feature reduction techniques, which reduced the number of probes to several hundred. We removed highly correlated probes. The univariate screen provided us with a list of 20 probes, 6 of which were significantly associated with the slope and remained significant after 10-fold cross-validation. Linear mixed model, with family structure as a random effect, showed that no family membership is a significant factor for this dynamics.

We could not explain the potential association of all found genes with dynamics of HTN incidence. Interestingly, however, 2 of these genes were previously associated with HTN in the available literature. *NOX3* is one of the isoforms of nicotinamide adenine dinucleotide phosphate (NADPH) oxidase, an enzyme-producing reactive oxygen species associated with renal function and a risk of diabetic nephropathy and HTN in Africans [8]. Recently, type I interferon, which mediates its effects through interferon-alpha receptor-1 gene *(IFNAR1)* was implicated in pulmonary HTN [9]. It is not known whether this gene may also have a role in systemic HTN, especially in diabetic patients.

These results encourage us to claim that gene expression data generated from PBMCs may be with careful modeling strategy associated with events distant in time.

## Conclusions

We think that our model is useful for analyzing combined transcriptomic and genomic data in complex diseases such as asthma, allergies, cancer, and obesity. Our strategy demonstrates using a modest-size phenotypic dataset to find associations with gene expression. We used a slope (regression coefficient) as an unbiased proxy for the blood pressure changes during development of the complex trait like hypertension. Apart from environmental factors, which are still the biggest causative agent of hypertension, we found genes that might be useful in predicting the progression of this disease.
